# Renal X-inactivation in female individuals with X-linked Alport syndrome primarily determined by age

**DOI:** 10.3389/fmed.2022.953643

**Published:** 2022-10-20

**Authors:** Roman Günthner, Lea Knipping, Stefanie Jeruschke, Robin Satanoskij, Bettina Lorenz-Depiereux, Clara Hemmer, Matthias C. Braunisch, Korbinian M. Riedhammer, Jasmina Ćomić, Burkhard Tönshoff, Velibor Tasic, Nora Abazi-Emini, Valbona Nushi-Stavileci, Karin Buiting, Nikola Gjorgjievski, Ana Momirovska, Ludwig Patzer, Martin Kirschstein, Oliver Gross, Adrian Lungu, Stefanie Weber, Lutz Renders, Uwe Heemann, Thomas Meitinger, Anja K. Büscher, Julia Hoefele

**Affiliations:** ^1^Department of Nephrology, Klinikum rechts der Isar, Technical University of Munich, School of Medicine, Munich, Germany; ^2^Institute of Human Genetics, Klinikum rechts der Isar, Technical University of Munich, School of Medicine, Munich, Germany; ^3^Pediatric Nephrology, University Hospital Essen, Essen, Germany; ^4^Molecular Epidemiology, Helmholtz Zentrum München, Munich, Germany; ^5^Department of Pediatrics I, University Children’s Hospital Heidelberg, Heidelberg, Germany; ^6^University Children’s Hospital, Medical Faculty of Skopje, Skopje, North Macedonia; ^7^Pediatric Clinic, University Clinical Center of Kosovo, Pristina, Kosovo; ^8^Institute for Human Genetics, University Hospital Essen, Essen, Germany; ^9^University Hospital of Nephrology, Faculty of Medicine, University “Ss Cyril and Methodius,” Skopje, North Macedonia; ^10^PHI SYNLAB Skopje Laboratories, Skopje, North Macedonia; ^11^Department of Pediatrics, Children’s Hospital St. Elisabeth and St. Barbara, Halle (Saale), Germany; ^12^Department of Pediatrics, General Hospital, Celle, Germany; ^13^Clinic for Nephrology and Rheumatology, University Medical Center Göttingen, Göttingen, Germany; ^14^Fundeni Clinical Institute, Pediatric Nephrology Department, Bucharest, Romania; ^15^Department of Pediatrics II, University Children’s Hospital, Philipps-University Marburg, Marburg, Germany

**Keywords:** Alport syndrome, X-inactivation, *COL4A5*, urine-derived cells, microscopic hematuria, proteinuria, end-stage kidney disease

## Abstract

X-linked Alport syndrome (AS) caused by hemizygous disease-causing variants in *COL4A5* primarily affects males. Females with a heterozygous state show a diverse phenotypic spectrum ranging from microscopic hematuria to end-stage kidney disease (ESKD) and extrarenal manifestations. In other X-linked diseases, skewed X-inactivation leads to preferential silencing of one X-chromosome and thus can determine the phenotype in females. We aimed to show a correlation between X-inactivation in blood and urine-derived renal cells and clinical phenotype of females with a heterozygous disease-causing variant in *COL4A5* compared to healthy controls. A total of 56 females with a heterozygous disease-causing *COL4A5* variant and a mean age of 31.6 ± 18.3 SD years were included in this study. A total of 94% had hematuria, 62% proteinuria >200 mg/day, yet only 7% had decreased eGFR. Using human androgen receptor assay X-inactivation was examined in blood cells of all 56 individuals, in urine-derived cells of 27 of these individuals and in all healthy controls. X-inactivation did not correlate with age of first manifestation, proteinuria or eGFR neither in blood, nor in urine. The degree of X-inactivation showed a moderate association with age, especially in urine-derived cells of the patient cohort (*rho* = 0.403, *p* = 0.037). Determination of X-inactivation allelity revealed a shift of X-inactivation toward the *COL4A5* variant bearing allele. This is the first study examining X-inactivation of urine-derived cells from female individuals with AS. A correlation between phenotype and X-inactivation could not be observed suspecting other genetic modifiers shaping the phenotype in female individuals with AS. The association of X-inactivation with age in urine-derived cells suggests an escape-mechanism inactivating the *COL4A5* variant carrying allele in female individuals with AS.

## Introduction

Alport syndrome (AS) is a hereditary nephropathy characterized by (microscopic) hematuria, proteinuria, chronic kidney disease potentially progressing to end-stage kidney disease (ESKD), hearing loss, and typical ocular changes (1, 2). The syndrome is caused by disease-causing [(likely) pathogenic] variants in genes encoding collagen type IV leading to an altered glomerular basement membrane (GBM). AS can be inherited in an X-linked form due to disease-causing variants in *COL4A5* or by disease-causing variants in *COL4A3* or *COL4A4* comparable with an autosomal inheritance (3–9).

Males with X-linked AS show a distinct genotype-phenotype correlation, illustrated by more severe symptoms in individuals with loss-of-function variants and less severe symptoms in individuals with missense variants or in-frame deletions. Additionally, variants located at the 5′ end have been described to present with earlier onset of ESKD and higher risk of extrarenal manifestations (10, 11).

Female individuals with heterozygous disease-causing variants in *COL4A5* have traditionally been described as healthy carriers even though the penetrance is variable both intrafamilial and interfamilial, showing a broad spectrum of clinical symptoms, varying from mild isolated microscopic hematuria to severe AS (12–15). By age 60 years, 15–30% of these female individuals develop ESKD. Furthermore, extrarenal manifestation like eye abnormalities and hearing impairment have been described and can affect up to 30% of female individuals (13). Until now, the cause for the phenotypic variability in female individuals with a heterozygous disease-causing variant in *COL4A5* is unclear. In contrast to male individuals, genotype-phenotype correlation is less-well described in females so far but severe variants (e.g., loss-of-function variants) appear to result in a more severe phenotype–similarly to affected males (13, 16).

Interindividual variability in females with a heterozygous disease-causing *COL4A5* variant might be explained by skewed, i.e., preferential X-inactivation of one X chromosome, as already discussed in the literature (12, 17, 18). X-inactivation results in transcriptional silencing of one X chromosome in females to attain gene dosage parity between XX female and XY male mammals (19). Either the maternal or the paternal X chromosome is randomly silenced through a complex cellular process resulting in a female being a mosaic of cells with either an active maternal or paternal X chromosome (17). X-inactivation has long been thought to be an irreversible occurrence. In recent years, there is increasing evidence, that in cells with rapid turnover, such as peripheral blood cells and buccal cells, skewing of X-inactivation increases with older age (20–23). However, an age-dependent mechanism in other tissues and their possible implications have not been shown yet.

So far, only insufficient molecular genetic investigations can be found focusing on X-inactivation in AS. Vetrie et al. tried to detect a correlation between the X-inactivation allelity of female carriers and the severity of their disease, but in DNA derived from peripheral blood lymphocytes no correlation was found (17). In contrast, Rheault et al. could show that X-inactivation is a major modifier of the female carrier phenotype in murine X-linked AS (24).

In this study, the relationship of X-inactivation of female individuals with a heterozygous disease-causing *COL4A5* variant with phenotypic characteristics in blood as well as urine-derived cells consisting of podocytes and tubular cells was investigated. Furthermore, these X-inactivation data were compared with those from healthy controls.

## Materials and methods

### Study population and design

Inclusion criteria of the study were proof of a heterozygous disease-causing *COL4A5* variant and biologically female gender. Individuals of all ages were allowed to be included in the study (age range of included individuals: 5–79 years). There were no comorbidities observed in the included individuals. Exclusion criteria were missing written informed consent and absence of above-mentioned inclusion criteria. Recruitment was primarily achieved by acquiring relatives of known male AS individuals with disease-causing *COL4A5* variants. Altogether 60 female individuals were recruited fulfilling the inclusion criteria. Four individuals had to be excluded due to indistinguishable X-inactivation analysis (see subsection X-inactivation analysis), leading to 56 available individuals from 43 unrelated families. In 49 of those individuals, a blood sample from an AS-affected relative was available needed for determining the potential skewing.

A total of 43/56 patients were recruited as part of the NephroGen cohort of the Institute of Human Genetics at the Klinikum rechts der Isar of the Technical University of Munich, and 2/56 at the Department of Pediatrics I of the University Children’s Hospital in Heidelberg. The remaining 11 patients were recruited at the Department of Pediatric Nephrology of the University Hospital in Essen, Germany. The study was approved by the respective local ethics committee of each contributing center and performed in accordance to the standards of the 2013 Helsinki Declaration. All individuals or their legal guardians gave written and informed consent.

### Phenotypic characteristics

Phenotypic information was gathered by studying medical records and interviews. Age of first manifestation was considered as time point of first proof of an abnormal renal phenotype (either microscopic hematuria, macroscopic hematuria or proteinuria >200 mg/day) or diagnosis of an extrarenal manifestation. When 24-h urine collection was not available, urinary protein/creatinine ratio of more than 200 mg/g creatinine was considered as proteinuria. Semi-quantitative urine dipstick measurements with the result “negative” were considered as proteinuria less than 200 mg/day. Serum creatinine values were determined by the treating physician. eGFR was calculated for individuals >14 years with CKD-EPI formula (25), for individuals ≤14 years with the “Bedside Schwartz formula” (26) considering the height and serum creatinine of the individual.

Healthy female control individuals without history of AS in their families were recruited and blood was drawn as well as spontaneous urine collected. Out of 40 healthy control individuals, five were homozygous for the AR locus and therefore not useable for the control cohort of this study. X-inactivation data of the remaining 35 female healthy controls were available for the study.

### Genotype characteristics

Supplementary Table 1 displays *COL4A5* variants and the clinical phenotype of the affected individuals. Variants were adjusted to the RefSeq Sequence NM_033380.3. Frameshift, non-sense and canonical splice site variants as well as a large deletion (70 kb, exons 38–51) were considered loss-of-function variants. For statistical calculation, two variants with small in-frame deletions were grouped with missense variants (c.2048_2050delCTG, p.Pro683_Gly684delinsArg, and c.1751_1756delCAGGGC, p.Pro584_Gly585del). Additionally, they were counted as glycine-affecting variants.

The severity of variants was calculated based on a classification of variants according to their position in the transcript. Variants were not located in the signal peptide and NC2 domain. Variants were classified according to their domain and the proximity to the 5′-end. Variants in the 5′-end near collagenous domain (c.124-c.2246) were considered “severe,” variants in the 3′-end near collagenous domain (c.2247-c.4386) were considered “moderate” and variants in the NC1 domain (c.4399-c.5073; 3′-end part of the protein) were considered “mild” (Supplementary Table 2) (10).

### Blood and urine cell DNA isolation

DNA was automatically extracted from EDTA blood samples with “Chemagic DNA Blood 5 k Kit” using a Chemagen 360 (PerkinElmer, Waltham, MA, USA) according to the manufacturer’s instructions.

Kidney tissue from biopsy samples is the gold standard to obtain renal-derived cells but was seldom available for analysis. Therefore, we compared X-inactivation in tissue-derived and urine-derived cells from individuals without renal disease and achieved comparable (less than 20% difference) results in 75% of individuals.

In addition, urine-derived cells were analyzed in five individuals with AS of the study cohort regarding the presence of podocytes and tubular cells. Therefore, podocytes and tubular cells were isolated from urine samples with magnetic cell separation using the “CD10 Microbead Kit human” (Miltenyi Biotec B.V. & Co.KG, Bergisch Gladbach, Germany). Subsequent FACS analysis confirmed the enrichment of CD10 positive cells. DNA was then extracted from these cells with Qiagen’s “DNeasy Blood and Tissue Kit 250” (Qiagen GmbH, Hilden, Germany).

The expression of CD10 in podocytes and tubular cells has been demonstrated in immunohistochemical stainings (Supplementary Figure 1). In 80% of analyses both cell types were detected within the urinary sediment. For these analyses first morning urine was collected and urine samples were immediately centrifuged for 10 min at 14,000 rpm at 4°C. Afterward the supernatant was discarded and urine cells were isolated right away or immediately frozen and stored at −80°C (for DNA isolation).

### X-inactivation analysis

The X-inactivation status was measured by assessing the DNA methylation based on the difference between the number of trinucleotide repeats at the human androgen receptor locus (*AR*, Xq11-12; HUMARA-Assay) (21).

Due to the correlation of X-inactivation with hypermethylation, digestion with methylation sensitive restriction enzymes (*Hpall*) can differentiate between active and inactive alleles. A male sample was enclosed in each assay in order to control sufficient *Hpall* digestion. Subsequent to digestion and amplification of the samples with fluorescence-tagged PCR primers, fragment length analysis of the PCR products was run on an ABI 3130XL genetic analyzer and the GeneMarker software (Softgenetics, PA, USA). If amplified DNA fragments exhibited the same size on both alleles, X-inactivation could not be determined.

X-inactivation data was generated and is displayed in two different ways depending on the availability of an affected male relative. In a first step, skewness of X-inactivation was measured with the ratio of inactivation between the two alleles without knowledge which of the alleles carried the *COL4A5* variant (e.g., 80:20 or 72:28). These data are referred to as “X-inactivation without allelity” and are displayed in a range from theoretically 50% (not skewed, both alleles with same X-inactivation percentage) to 100% (completely skewed).

In families with an available affected male, segregation analysis of the male individual (0% X-inactivation, *COL4A5* variant bearing allele not inactivated at all) allowed determining which of the two alleles in a female individual with a heterozygous disease-causing variant was the one bearing the *COL4A5* variant (27). Thus X-inactivation data could be displayed in a range from theoretically 0% (*COL4A5* variant bearing allele not inactivated at all) to 100% (*COL4A5* variant bearing allele completely inactivated). This dataset is referred to as “X-inactivation with allelity.”

### Statistical analysis

Parametric data are presented as mean ± standard deviation and non-parametric data as median [Interquartile range, IQR]. For correlation analyses Spearman’s rho was calculated (Figures 2, 3 and Table 1). When comparing two groups (Table 2 and Supplementary Table 3) we used Mann–Whitney *U*-test for non-parametric variables, Student’s *t*-test for parametric variables and Chi square test for categorical variables. For comparison of more than 2 groups Kruskal–Wallis test was applied due to the non-normal distribution of the dependent parameters (Supplementary Table 2). SPSS^®^ Statistics, version 26 (IBM, Armonk, NY, USA), was used for all statistical tests.

**TABLE 1:** Correlation coefficients of X-inactivation in blood and urine with phenotypic markers in female individuals with a heterozygous variant in *COL4A5.*

		X-inactivation urine without allelity	X-inactivation urine with allelity	X-inactivation blood without allelity	X-inactivation blood with allelity
Age at analysis	*rho*	**0.403**	0.348	0.104	0.202
	*p*-value	**0.037**	0.076	0.444	0.165
	*n*	27	27	56	56

Age of first manifestation	*rho*	0.139	0.137	−0.084	0.051
	*p*-value	0.507	0.513	0.548	0.737
	*n*	25	25	53	46

eGFR	*rho*	−0.032	−0.110	−0.298	−0.043
	*p*-value	0.895	0.655	0.056	0.797
	*n*	19	19	42	38

Proteinuria >200 mg/day	*rho*	−0.213	−0.017	0.103	0.192
	*p*-value	0.307	0.937	0.462	0.19
	*n*	25	25	53	48

X-inactivation without allelity: The ratio of inactivation was measured between the two alleles without knowledge which of the alleles carried the *COL4A5* variant. X-inactivation with allelity: The ratio of inactivation was measured between the two alleles with knowledge which of the alleles carried the *COL4A5* variant. Bold values indicate *p*-value < 0.05.

**TABLE 2:** Phenotypic characteristics and type of variant.

*COL4A5* variants	Loss-of-function (*n* = 24)	Missense (*n* = 32)	*P*-value
Any manifestation, in %	95.8% (23/24)	93.8% (30/32)	0.732
Age of first manifestation, years, median [IQR]	6.6 [3.9; 10.8]	12.4 [6.5; 27.8]	**0.011**
Proteinuria >200 mg/day^§^, in %	65.2% (15/23)	60.0% (18/30)	0.698
eGFR, ml/min/1.73 m^2^, mean ± SD^§^	103 ± 24	101 ± 29	0.775
End-stage kidney disease, in %	4.2% (1/24)	9.4% (3/32)	0.454
Ocular manifestation^§§^, in %	12.5% (3/24)	3.1% (1/32)	0.178
Hearing impairment^§§^, in %	8.3% (2/24)	3.1% (1/32)	0.392

^§^Differing patient numbers: eGFR (*n* = 42), proteinuria (*n* = 53). ^§§^Affected individuals were examined by a specialized ophthalmologist and otologist. Bold values indicate *p*-value < 0.05.

## Results

### Demographics and phenotype/genotype of individuals

**FIGURE 1:**
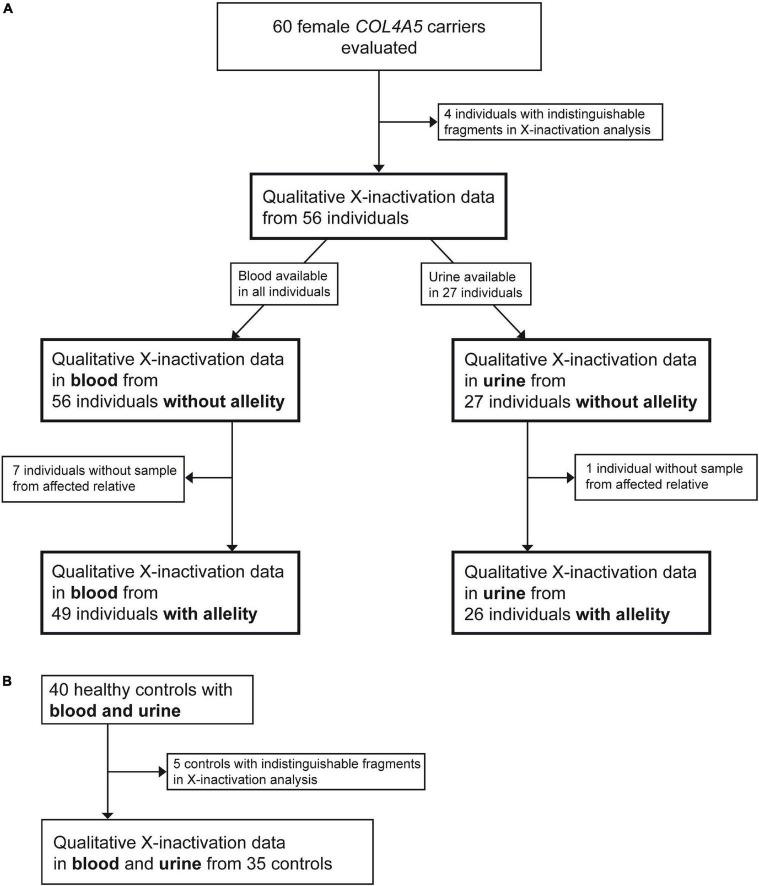
Flowchart illustrating the recruitment process of participants. Figure shows recruitment procedure for individuals with a heterozygous disease-causing *COL4A5* variant **(A)** and healthy controls **(B)**.

In total, 56 resp. 27 female individuals with a disease-causing variant in *COL4A5* with X-inactivation data in peripheral blood cells resp. in urine-derived cells were available for analysis (Figure 1). In 49 of the 56 blood X-inactivation datasets, the *COL4A5*-dependent allelity of X-inactivation was revealed by analyzing affected male relatives. For the urine-derived samples, *COL4A5*-dependent X-inactivation allelity could be determined in 26 out of 27 samples (Figure 1).

The mean age of the included individuals was 31.6 ± 18.3 years (mean ± SD; Table 3). Almost every female individual with a heterozygous disease-causing *COL4A5* variant had a renal manifestation (94.6%) with first symptoms/abnormal urine analysis detected at a median age of 9.5 [IQR 5.3; 19.7] years. Most common manifestation was hematuria (93.7%; microscopic or macroscopic) followed by proteinuria >200 mg/day (62.3%), whereas mean eGFR was normal. Four of the 56 individuals progressed to ESKD with a mean age of onset at 32.8 ± 5.3 years (mean ± SD). Extrarenal manifestation defined as hearing impairment (5.4%) or ocular manifestation (7.1%) were detected only in a few individuals.

**TABLE 3:** Phenotype and genotype of individuals for total and urine-analysis containing cohort.

Demographics and phenotype	Total cohort (*n* = 56)	Urine analysis available (*n* = 27)
Age at analysis, years, mean ± SD	31.6 ± 18.3	34.7 ± 19.9
Any manifestation, in %	94.6% (53/56)	926% (25/27)
First manifestation, age in years, median [IQR]	9.5 [5.3; 19.7]	10.0 [5.5; 18.1]
Hematuria, in %	93.7% (52/56)	92.6% (25/27)
Microscopic hematuria, in %	82.1% (46/56)	85.2% (23/27)
Macroscopic hematuria, in %	10.7% (6/56)	7.4% (2/27)
Proteinuria >200 mg/day, in %	62.3 (33/53)	56.0% (14/25)
Proteinuria quantified per day, median [IQR]^§^	0.34 [0.17; 0.80]	0.29 [0.13; 0.49]
eGFR, ml/min/1.73 m^2^, mean ± SD^§^	101.7 ± 26.9	89 ± 27
End-stage kidney disease, in %	7.1% (4/56)	0%
Age of onset ESKD, years, mean ± SD	32.8 ± 5.3	–
Renal transplant, in %	1.8% (1/56)	–
Hearing impairment, in %	5.4% (3/56)	11.1% (3/27)
Ocular manifestation, in %	7.1% (4/56)	7.4% (2/27)
**Genotype**		
LoF variant, in %	42.9% (24/56)	51.9% (14/27)
Frameshift, in %	21.4% (12/56)	29.6% (8/27)
Nonsense, in %	3.6% (2/56)	7.4% (2/27)
Exon-spanning deletion, in %	3.6% (2/56)	–
Splice, in %	14.3% (8/56)	14.8% (4/27)
Missense variant^§§^, in %	57.1% (32/56)	48.1% (13/27)
Glycine, in %	51.8% (29/56)	40.7% (11/27)
Non-glycine, in %	5.4% (3/56)	7.4% (2/27)
**Severity of variant (Bekheirnia et al.)**		
5′-end, in %	3.6% (2/56)	3.7% (1/27)
Collagenous, in %	87.5% (49/56)	77.8% (21/27)
3′-end, in %	8.9% (5/56)	18.5% (5/27)

^§^Differing individual numbers. Urine cohort: eGFR (*n* = 19), proteinuria quantified (*n* = 11); blood cohort: eGFR (*n* = 42), proteinuria quantified (*n* = 38). ^§§^Includes two in-frame indel mutations. LoF; loss-of-function; ESKD; end-stage kidney disease.

Loss-of-function variants were present in 42.9% of cases, mostly represented by frameshift and canonical splice site variants (Table 3). Individuals with missense variants had primarily glycine-variants except for 3 out of 32 individuals. Most variants were located in the collagenous domain of the *COL4A5* gene (87.5%).

Comparing the total cohort of 56 individuals with the 27 individuals with additional urine samples, the subcohort of individuals with X-inactivation data from urine-derived cells were slightly older, had less proteinuria, slightly decreased eGFR and did not include individuals with ESKD due to loss of remaining urine excretion or history of kidney transplantation (Table 3).

Healthy controls (*n* = 35) had no history of AS in the family, were all female and had a mean age of 37.1 ± 19.1 years (mean ± SD).

### Age of first manifestation in females with a disease-causing *COL4A5* variant depends on variant type

Median age of first manifestation was significantly lower in individuals with loss-of-function variants compared to individuals with missense variants (Table 2) (6.6 vs. 12.4 years, *p* = 0.011). Individuals with ocular manifestations or hearing impairment predominately carried loss-of-function variants.

After classification of variants according to their expected severity (10), most variants were located in the collagenous domain, which can be divided in the more severe 5′-end near part and the less severe 3′-end near part (Supplementary Table 2). Individuals with variants near the 5′-end showed earlier first manifestation, higher percentage of proteinuria >200 mg/day, ocular manifestation and ESKD, yet without significance. The variants of the four individuals who progressed to ESKD were three missense and one splice site variant.

### X-inactivation shows strong association between blood and urine-derived cells

Median X-inactivation of cases without considering allelity was 65% [IQR 58; 72] in blood cells and 59% [IQR 53; 69] in urine-derived cells (data not shown). Analysis of *COL4A5*-dependent X-inactivation of blood and urine samples showed a tendency toward inactivation of the variant bearing allele (55% and 56%). For the controls, the degree of X-inactivation amounted to median 65% [IQR 59; 79] for blood cells and 66% [IQR 59; 70] for urine-derived cells.

**FIGURE 2:**
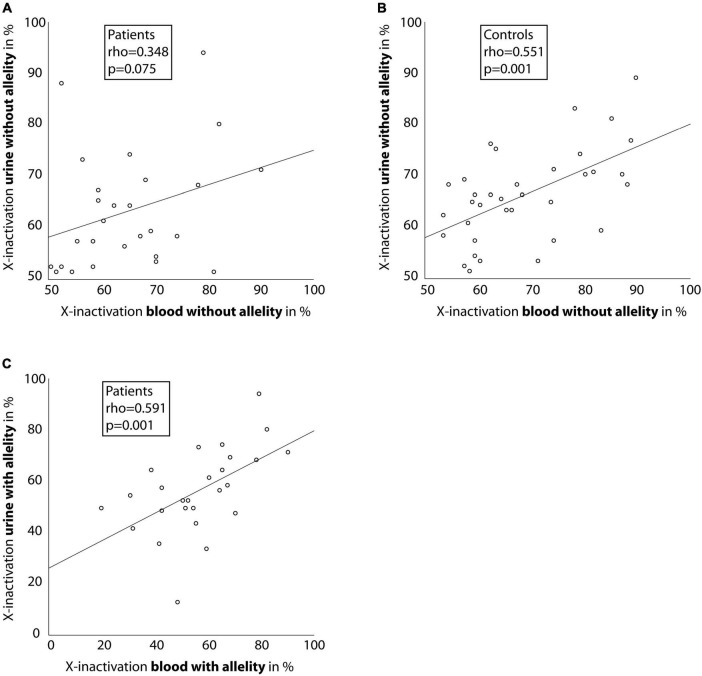
Correlation between X-inactivation in blood and urine cells. Scatter-dot plots of the relationship of X-inactivation between blood and urine cells for individuals with a heterozygous disease-causing *COL4A5* variant **(A,C)** and healthy controls **(B)**. Spearman’s rho was calculated and is displayed in the boxes. Linear regression lines were plotted for illustration purposes.

X-inactivation measured without allelity showed a medium to strong association between blood and urine-derived cells in affected female individuals and healthy controls (*rho* = 0.348, *p* = 0.075 and *rho* = 0.551, *p* = 0.001; Figures 2A,B), however the association was not significant in affected individuals. Regarding the *COL4A5*-dependent X-inactivation allelity, the association between blood and urine-derived cells was even higher (*rho* = 0.591, *p* = 0.001; Figure 2C).

### Age determines X-inactivation of female individuals with disease-causing *COL4A5* variants especially in urinary cells

**FIGURE 3:**
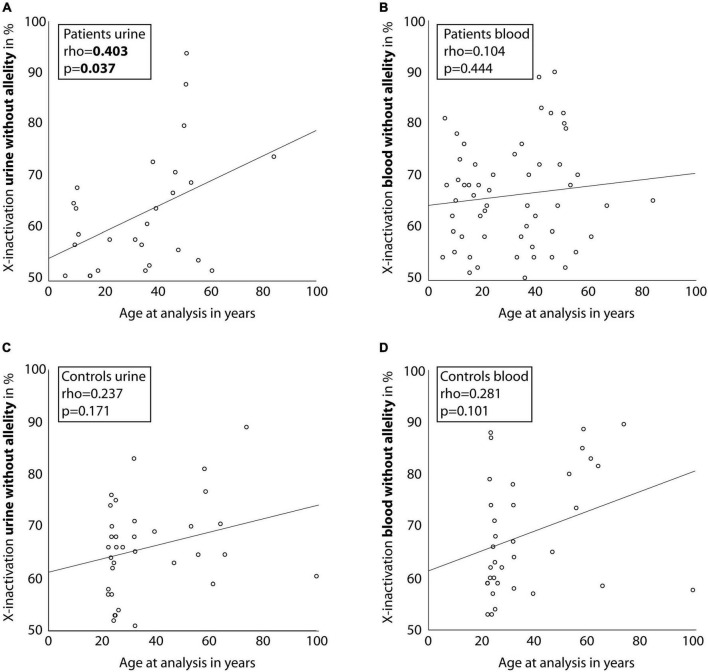
Correlation between age of individuals and X-inactivation in blood and urine cells. Scatter-dot plots of the association of participant age and X-inactivation in blood and urine cells for individuals with a heterozygous disease-causing *COL4A5* variant **(A,B)** and healthy controls **(C,D)**. Spearman’s rho was calculated and is displayed in the boxes. Linear regression lines were plotted for illustration purposes.

Correlating age with X-inactivation (without allelity) in urine-derived cells of the affected individuals, a significant association with a Spearman’s rho of 0.403 (*p* = 0.037, Figure 3A) was seen. Considering X-inactivation with *COL4A5*-dependent allelity, the association with age was similar (*rho* = 0.348, Supplementary Figure 2A), however not significant. In comparison, X-inactivation in blood cells of affected individuals showed weak, non-significant correlations with age, with or without considering the *COL4A5*-dependent allelity (Figure 3B and Supplementary Figure 2B).

Comparing the individuals to healthy controls, the latter showed only a weak association of X-inactivation with age for both, blood and urinary cells (Figures 3C,D; *rho* = 0.237; *p* = 0.171, and *rho* = 0.281; *p* = 0.101; Supplementary Figure 3).

### Phenotype and X-inactivation in female individuals with heterozygous variants causing X-linked Alport syndrome

Correlation analyses of phenotypic characteristics and X-inactivation could not demonstrate a significant association for age of first manifestation, eGFR or proteinuria (Table 1). There was a trend toward decreased eGFR with enhanced X-inactivation, however eGFR and age naturally showed a strong dependency in our cohort (*rho* = −0.547, *p* < 0.001; data not shown).

Further analysis of blood of individuals with ESKD (*n* = 4) revealed increased X-inactivation without allelity (77% in ESKD vs. 64% in non-ESKD) as well as increased X-inactivation with *COL4A5*-dependent allelity (72% in ESKD vs. 55% in non-ESKD, Supplementary Table 3).

Individuals with ocular manifestation or hearing impairment showed increased X-inactivation in urine-derived cells. X-inactivation with and without allelity was 71% in individuals with ocular manifestations vs. 53–58% in individuals without ocular manifestations. Regarding hearing impairment, the results were similar (69–73% in individuals affected by hearing impairment vs. 54–58% without hearing impairment; Supplementary Table 3). However, blood cells did not show differences in X-inactivation when comparing individuals with and without extrarenal manifestations. Of the 5 individuals presenting with extrarenal manifestations, everyone had normal eGFR, but four individuals exhibited proteinuria >200 mg/day with one individual presenting with proteinuria of 1.42 g/day. However, statistical analysis was not possible due to the small number of individuals with ESKD or extrarenal manifestations.

## Discussion

This is the first study investigating X-inactivation in urine-derived cells of female individuals with X-linked AS. Additionally, the *COL4A5*-dependent allelity of X-inactivation depending on the *COL4A5* variant bearing allele was included in the analysis as a novelty in a human AS cohort.

Unlike the hypothesis, X-inactivation was not relevantly associated with phenotypic characteristics like age of first manifestation, proteinuria or decreased eGFR. However, we were able to demonstrate a statistically significant correlation of age with skewed X-inactivation in urine-derived cells of female individuals with AS. This correlation was also found in peripheral blood cells of the individuals as well as in urine-derived and blood cells of the healthy controls, yet only in a weak, not statistically significant fashion.

The variability of X-inactivation and its association with age has been proven in cross-sectional and recently in longitudinal studies enrolling healthy controls (22, 28, 29). The mechanisms for this association are unclear, but intrinsic as well as environmental influences have been suggested (28). So far, correlation of X-inactivation with age has only been demonstrated for rapidly proliferating tissues like blood granulocytes or buccal epithelium (21, 23). A recent study including over 300 healthy females could not show an age-dependent effect in slowly proliferating cells like fat or skin tissue, but it did show an effect in lymphocyte-derived cells (22). The only study investigating cells isolated from urine of approximately 40 healthy controls was not able to show an age-dependent effect of X-inactivation (21). Our results in healthy controls show only a weak, non-significant correlation of age with X-inactivation in urine-derived cells, which underlines the observation for slowly proliferating cells in the existing literature.

In contrast, urine-derived cells from our individuals with AS exhibited a moderate and statistically significant correlation of age with X-inactivation. Older individuals with AS were also more likely to present with increased X-inactivation of the variant allele (*rho* = 0.348, *p* = 0.076) and the degree of X-inactivation of the disease-causing allele amounted to 55% in blood cells and 56% in urine-derived cells. This suggests that X-inactivation could serve as an escape-mechanism protecting individuals from the development of severe complications of AS as they get older. Involvement of X-inactivation has been described for heterozygous carriers in X-linked diseases like severe combined immunodeficiency (30) and X-linked intellectual disability (31). In these individuals the allelity of X-inactivation also led to a more inactive disease variant bearing allele in heterozygous carriers. Thus, the gene dosage is adjusted in favor of the wild-type allele by silencing the mutant allele. The proposed mechanism for this phenomenon is a survival selection of cells which have inactive disease-causing alleles. Even though the data might suggest that this is also the case in individuals with a heterozygous disease-causing variant in *COL4A5*, the size of the present study cohort is not sufficient to definitely draw this conclusion. Further studies including more individuals with a severe phenotype would be needed to elucidate these mechanisms.

Regarding the relationship between the tissues, the data showed a moderate to high and significant correlation between X-inactivation in blood and urine-derived cells (*rho* = 0.551, *p* = 0.001 for controls; and *rho* = 0.591, *p* = 0.001 for cases considering *COL4A5*-dependent X-inactivation allelity). These associations were stronger than described in the literature for cells from other slowly proliferating tissues (like fat and skin) (22). As blood cells are known to have the highest degrees of X-inactivation skewness in healthy subjects compared to other tissues (22, 23), urine cells should be considered when analyzing X-inactivation in certain cases.

Nonetheless, the hypothesized X-inactivation-phenotype relationship could not be shown in blood or urine-derived cells. This is in line with the previous observation in 30 female individuals with a heterozygous disease-causing variant in *COL4A5* in blood cells only (17). Murine data, however, point to a role for X-inactivation in phenotype development in an AS mouse model (24). X-inactivation of the mutant allele was correlated with less proteinuria and plasma urea nitrogen, demonstrated in cells from whole murine kidneys. Yet one has to keep in mind that X-inactivation is measured differently in mice, and the effect of age, which evidently plays an important role in human X-inactivation, is not accounted for. Additionally, a standardized inbred murine model cannot be compared with humans, especially as more evidence emerges demonstrating a role for other genetic modifiers influencing the phenotype like *FMN1*, which has been described to be associated with albuminuria in *COL4A5*-deficient mice (32). In general, the genotype-phenotype correlation in female individuals with a heterozygous disease-causing *COL4A5* variant is weak compared to descriptions in male individuals with AS (16). A slight, but significant difference in age of first manifestation between loss-of-function and missense variants could be seen, however, no difference regarding renal parameters relevant for prognosis were found (proteinuria, eGFR). As X-inactivation does not seem to play a big role in female individuals with AS apart from age, other genetic modifiers like disease-causing variants or single nucleotide polymorphisms in other genes like autosomal *COL4A3*, *COL4A4* or recently described *FMN1* become increasingly important and future studies should be dedicated to these (32).

One limitation of the present study is the relatively young age of included individuals (31.6 years), which also led to decreased numbers of individuals with ESKD and extrarenal manifestations. This bias is based on the recruitment process, which mostly included sisters or mothers of pediatric patients. The degree of skewness in blood cells evidently accelerates above the age of 60 years (22). Thus, associations of X-inactivation with phenotypic characteristics could have been detected with an older study population. Additionally, our study population primarily included individuals of Caucasian descent and therefore the results might not be applicable for all ethnicities. Another limitation of the study applies to the method of X-inactivation measurement. Indistinguishable PCR fragments due to same-sized trinucleotide repeats can make an analysis impossible as it was the case in nine participants of this study. Moreover, collection of urine-derived cells in individuals with ESKD is difficult due to loss of urine production or kidney transplantation, which often excludes severely affected individuals from X-inactivation analysis of urine-derived cells. Renal cell isolation and transportation (dry ice) is also more complex than drawing EDTA-blood resulting in a markedly reduced number of urine X-inactivation data.

In conclusion, our data show an association of X-inactivation and age in urine-derived cells of female individuals with AS, which could present an escape-mechanism to avoid expression of the *COL4A5* variant as individuals get older. A relationship between X-inactivation and phenotypic characteristics was not present in blood as well as urine cells. Future studies about X-inactivation in female individuals should focus on older cohorts.

## Data availability statement

The datasets presented in this study can be found in online repositories. The names of the repository/repositories and accession number(s) can be found in the article/Supplementary material.

## Ethics statement

This study was approved by the respective local ethics committee of each contributing center. Written informed consent to participate in this study was provided by the participants’ legal guardian/next of kin. Written informed consent was obtained from the individual(s), and minor(s)’ legal guardian/next of kin, for the publication of any potentially identifiable images or data included in this article.

## Author contributions

RG, LK, SJ, BL-D, CH, KR, JC, and KB performed the analysis. RS, MB, BT, VT, NA-E, VN-S, NG, AM, LP, MK, OG, AL, SW, LR, UH, TM, and AB cared for the patients and acquired and provided the clinical data. RG, AB, and JH were responsible for writing and revision of the manuscript. All authors contributed to the article and approved the submitted version.
