# Supplementary material for: Renal X-inactivation in female individuals with X-linked Alport syndrome primarily determined by age
Source: Front Med (Lausanne). 2022 Oct 20;9:953643. doi: 10.3389/fmed.2022.953643 (PMC9630586; doi:10.3389/fmed.2022.953643)
Supplement: Supplementary file 1 [file Data_Sheet_1.PDF]

Renal X-inactivation in female individuals with X-linked Alport syndrome primarily determined by age

Supplementary Tables, Figures and Legends

Supplementary Table 1	2
Supplementary Table 2	3
Supplementary Table 3	4
Supplementary Figure 1	5
Supplementary Figure 2	6
Supplementary Figure 3	7
Supplementary Table and Figure Legends	8

Supplementary Table 1

Individual/Family	Gene	Transcript number	Nucleotide change	Amino acid change	ACMG rating	ACMG classification	Microscopic hematuria/ Macroscopic hematuria	Proteinuria > 200 mg/day	ESKD	Ocular manifestation	Hearing impairment
ATS-F173-II-2	*COL4A5*	NM_033380.3	c.1871G>A	p.(Gly624Asp)	PS4_moderate PM1_strong PP3	Likely pathogenic	None	No	No	No	No
ATS-F184-II-2	*COL4A5*	NM_033380.3	c.2300dup	p.(Lys768Glnfs*9)	PVS1 PM2	Likely pathogenic	Microscopic hematuria	Yes	No	No	No
ATS-F187-I-2	*COL4A5*	NM_033380.3	c.4763del	p.(Gln1588fs)	PVS1 PM1 PM2 PP4	Pathogenic	Microscopic hematuria	Yes	No	No	Yes
ATS-F187-II-2	*COL4A5*	NM_033380.3	c.4763del	p.(Gln1588fs)	PVS1 PM1 PM2 PP5	Pathogenic	Microscopic hematuria	Yes	No	No	No
ATS-F236-II-2	*COL4A5*	NM_033380.3	c.1976_1977del	p.(Ile659Asnfs*11)	PVS1 PM2	Likely pathogenic	Microscopic hematuria	No	No	No	No
ATS-F242-IV-1	*COL4A5*	NM_033380.3	c.2465G>T	p.(Gly822Val)	PS4_moderate PM1_strong PP3	Likely pathogenic	Macroscopic hematuria	N/A	Yes	No	No
ATS-F243-II-1	*COL4A5*	NM_033380.3	c.1871G>A	p.(Gly624Asp)	PS4_moderate PM1_strong PP3	Likely pathogenic	Microscopic hematuria	No	No	No	No
ATS-F249-II-1	*COL4A5*	NM_033380.3	c.1751_1756delCAGGGC	p.(Pro584_Gly585del)	PM4 PM1_strong PM2	Likely pathogenic	Macroscopic hematuria	Yes	Yes	No	No
ATS-F250-II-1	*COL4A5*	NM_033380.3	c.1673G>A	p.(Gly558Asp)	PS4_supporting PM1_strong PM2 PM5 PP3	Pathogenic	Macroscopic hematuria	Yes	No	No	No
ATS-F261-II-1	*COL4A5*	NM_033380.3	c.645+1G>T	p.(?)	PVS1_strong PM2	Likely pathogenic	Microscopic hematuria	Yes	Yes	No	No
ATS-F263-II-1	*COL4A5*	NM_033380.3	c.188dup	p.(Phe64Ilefs*16)	PVS1 PM2	Likely pathogenic	Microscopic hematuria	Yes	No	Yes	No
ATS-F267-II-1	*COL4A5*	NM_033380.3	Deletion of exons 38-51	-	PVS1 PS4_supporting PM2	Pathogenic	Microscopic hematuria	Yes	No	No	No
ATS-F267-II-2	*COL4A5*	NM_033380.3	Deletion of exons 38-51	-	PVS1 PS4_supporting PM2	Pathogenic	Microscopic hematuria	No	No	No	No
ATS-F274-III-14	*COL4A5*	NM_033380.3	c.2359G>A	p.(Gly787Arg)	PS2 PM1_strong PM2 PM5 PP3	Pathogenic	Microscopic hematuria	Yes	No	No	No
ATS-F295-I-2	*COL4A5*	NM_033380.3	c.611G>A	p.(Gly204Asp)	PS2 PM1_strong PM2 PM5 PP3	Pathogenic	Microscopic hematuria	Yes	Yes	No	No
ATS-F295-II-1	*COL4A5*	NM_033380.3	c.611G>A	p.(Gly204Asp)	PS2 PM1_strong PM2 PM5 PP3	Pathogenic	Microscopic hematuria	Yes	No	No	No
ATS-F295-II-2	*COL4A5*	NM_033380.3	c.611G>A	p.(Gly204Asp)	PS2 PM1_strong PM2 PM5 PP3	Pathogenic	Microscopic hematuria	Yes	No	No	No
ATS-F336-I-2	*COL4A5*	NM_033380.3	c.1799G>T	p.(Gly600Val)	PM1_strong PM2 PM5 PP3	Likely pathogenic	Microscopic hematuria	No	No	No	No
ATS-F486-I-2	*COL4A5*	NM_033380.3	c.225del	p.(Gln76Lysfs*79)	PVS1 PM2	Likely pathogenic	Microscopic hematuria	Yes	No	No	No
ATS-F513-I-2	*COL4A5*	NM_033380.3	c.3721G>T	p.(Gly1241Cys)	PS4_moderate PM1_strong PM2 PP3	Likely pathogenic	Microscopic hematuria	No	No	No	No
ATS-F513-I-3	*COL4A5*	NM_033380.3	c.3721G>T	p.(Gly1241Cys)	PS4_moderate PM1_strong PM2 PP3	Likely pathogenic	Microscopic hematuria	Yes	No	No	No
ATS-F513-II-1	*COL4A5*	NM_033380.3	c.3721G>T	p.(Gly1241Cys)	PS4_moderate PM1_strong PM2 PP3	Likely pathogenic	Microscopic hematuria	No	No	No	No
ATS-F519-I-2	*COL4A5*	NM_033380.3	c.4217-1G>A	p.(?)	PVS1 PM2	Likely pathogenic	Microscopic hematuria	No	No	No	No
ATS-F519-II-1	*COL4A5*	NM_033380.3	c.4217-1G>A	p.(?)	PVS1 PM2	Likely pathogenic	Macroscopic hematuria	No	No	No	No
ATS-F535-I-2	*COL4A5*	NM_033380.3	c.3685G>A	p.(Gly1229Ser)	PS4_moderate PM1_strong PM2 PM5 PP3	Pathogenic	Macroscopic hematuria	Yes	No	No	No
ATS-F618-I-2	*COL4A5*	NM_033380.3	c.5038C>T	p.(Arg1680*)	PVS1 PM2	Likely pathogenic	None	No	No	No	No
ATS-F632-I-2	*COL4A5*	NM_033380.3	c.761_762del	p.(Glu254Valfs*11)	PVS1 PM2	Likely pathogenic	Microscopic hematuria	N/A	No	No	No
ATS-F635-I-2	*COL4A5*	NM_033380.3	c.1402C>T	p.(Gln468*)	PVS1 PM2	Likely pathogenic	Microscopic hematuria	No	No	Yes	Yes
ATS-F636-II-2	*COL4A5*	NM_033380.3	c.3772G>A	p.(Gly1258Ser)	PM1_strong PM2 PP3	Likely pathogenic	Microscopic hematuria	No	No	No	No
ATS-F638-I-2	*COL4A5*	NM_033380.3	c.1807G>A	p.(Gly603Ser)	PS4_moderate PM1_strong PP3	Likely pathogenic	Microscopic hematuria	N/A	No	No	No
ATS-F7-II-1	*COL4A5*	NM_033380.3	c.1871G>A	p.(Gly624Asp)	PS4_moderate PM1_strong PP3	Likely pathogenic	Microscopic hematuria	Yes	No	No	No
ATS-F7-III-1	*COL4A5*	NM_033380.3	c.1871G>A	p.(Gly624Asp)	PS4_moderate PM1_strong PP3	Likely pathogenic	Microscopic hematuria	No	No	No	No
ATS-F724-II-1	*COL4A5*	NM_033380.3	c.646-2A>G	p.(?)	PVS1 PM2	Likely pathogenic	Microscopic hematuria	Yes	No	No	No
ATS-F737-II-1	*COL4A5*	NM_033380.3	c.687del	p.(Gly230Valfs*24)	PVS1 PM1_strong PM2	Pathogenic	Macroscopic hematuria	Yes	No	No	No
ATS-F743-II-1	*COL4A5*	NM_033380.3	c.937-1G>A	p.(?)	PVS1 PS4_moderate PM2	Pathogenic	Microscopic hematuria	Yes	No	Yes	No
ATS-F743-II-2	*COL4A5*	NM_033380.3	c.937-1G>A	p.(?)	PVS1 PS4_moderate PM2	Pathogenic	Microscopic hematuria	No	No	No	No
ATS-F743-II-3	*COL4A5*	NM_033380.3	c.937-1G>A	p.(?)	PVS1 PS4_moderate PM2	Pathogenic	Microscopic hematuria	No	No	No	No
ATS-F8-II-1	*COL4A5*	NM_033380.3	c.3197G>A	p.(Gly1066Asp)	PS4_moderate PM2 PM5_strong PP3 BP1	Likely pathogenic	Microscopic hematuria	Yes	No	No	No
ATS-F8-II-2	*COL4A5*	NM_033380.3	c.3197G>A	p.(Gly1066Asp)	PS4_moderate PM2 PM5_strong PP3 BP1	Likely pathogenic	Microscopic hematuria	No	No	No	No
ATS-F8-II-3	*COL4A5*	NM_033380.3	c.3197G>A	p.(Gly1066Asp)	PS4_moderate PM2 PM5_strong PP3 BP1	Likely pathogenic	Microscopic hematuria	No	No	No	No
ATS-F88-II-1	*COL4A5*	NM_033380.3	c.2048_2050del	p.(Pro683_Gly684delinsArg)	PS2 PM2 PM4	Likely pathogenic	Microscopic hematuria	Yes	No	No	No
ATS-F91-I-2	*COL4A5*	NM_033380.3	c.1871G>A	p.(Gly624Asp)	PS4_moderate PM1_strong PP3	Likely pathogenic	None	No	No	No	No
ATS-F94-I-2	*COL4A5*	NM_033380.3	c.2642G>T	p.(Gly881Val)	PS4_supporting PM1_strong PM2 PP5	Likely pathogenic	None	Yes	No	No	No
ATS-F94-II-1	*COL4A5*	NM_033380.3	c.2642G>T	p.(Gly881Val)	PS4_supporting PM1_strong PM2 PP5	Likely pathogenic	Microscopic hematuria	Yes	No	No	No
ATS-F825-II-1	*COL4A5*	NM_033380.3	c.2686G>A	p.(Gly896Ser)	PS4_moderate PM1_strong PM2 PM5	Pathogenic	Microscopic hematuria	Yes	No	No	No
E3	*COL4A5*	NM_033380.3	c.4882_4885del	p.(Pro1628Serfs*24)	PVS1 PM2	Likely pathogenic	Microscopic hematuria	Yes	No	No	No
E4	*COL4A5*	NM_033380.3	c.2472dupA	p.(Gly825Argfs*30)	PVS1 PS4_supporting PM2	Pathogenic	Microscopic hematuria	Yes	No	No	No
E5	*COL4A5*	NM_033380.3	c.2332G>A	p.(Gly778Ser)	PS4_moderate PM1_strong PM2 PM5	Pathogenic	Microscopic hematuria	Yes	No	No	No
E8	*COL4A5*	NM_033380.3	c.1340-5C>G	p.(?)	PM2	VUS	Microscopic hematuria	Yes	No	No	No
E9	*COL4A5*	NM_033380.3	c.1165G>A	p.(Gly389Arg)	PS4_moderate PM1_strong PM2 PM5	Pathogenic	Microscopic hematuria	Yes	No	No	No
E11	*COL4A5*	NM_033380.3	c.3446G>A	p.(Gly1149Asp)	PS4_supporting PM1_strong PM2 PM5	Likely pathogenic	Microscopic hematuria	Yes	No	No	No
E12	*COL4A5*	NM_033380.3	c.4793C>T	p.(Ser1598Phe)	PS4_moderate PM1 PM2 PM5	Likely pathogenic	Microscopic hematuria	No	No	No	No
E13	*COL4A5*	NM_033380.3	c.1871G>A	p.(Gly624Asp)	PS4_moderate PM1_strong PP3	Likely pathogenic	Microscopic hematuria	Yes	No	Yes	Yes
E14	*COL4A5*	NM_033380.3	c.4882_4885del	p.(Pro1628Serfs*24)	PVS1 PM2	Likely pathogenic	Microscopic hematuria	Yes	No	No	No
E16	*COL4A5*	NM_033380.3	c.4793C>T	p.(Ser1598Phe)	PS4_moderate PM1 PM2 PM5	Likely pathogenic	Microscopic hematuria	No	No	No	No
E18	*COL4A5*	NM_033380.3	c.2048delC	p.(Pro683Leufs*53)	PVS1 PM1 PM2	Pathogenic	Microscopic hematuria	Yes	No	No	No

Supplementary Table 2

	5' near collagenous domain (n=28)	3' near collagenous domain (n=21)	NC1 domain (n=7)	*p*-value
**Any manifestation**	93 % (26/28)	100% (21/21)	86% (6/7)	0.292
**First manifestation age, median, years**	8.4	9.5	10.4	0.793
**Proteinuria >200 mg/d[§]**	65% (17/26)	60% (12/20)	57% (4/7)	0.892
**End-stage kidney disease**	11% (3/28)	5% (1/21)	0% (0/7)	N/A
**Ocular manifestation**	14% (4/28)	0% (0/21)	0% (0/7)	N/A
**Hearing impairment**	7% (2/28)	0% (0/21)	14% (1/7)	N/A

Supplementary Table 3

	End-stage kidney disease, “yes” vs “no”	Ocular manifestation, “yes” vs “no”	Hearing impairment, “yes” vs “no”
**X-inactivation urine without direction, median§**	N/A	71% vs. 58%	73% vs. 58%
**X-inactivation urine with direction, median§**	N/A	71% vs. 53%	69% vs. 54%
**X-inactivation blood without direction, median§**	77% vs. 64%	66% vs. 65%	56% vs. 65%
**X-inactivation blood with direction, median§**	72% vs. 55%	60% vs. 55%	56% vs. 57%

Supplementary Figure 1

A

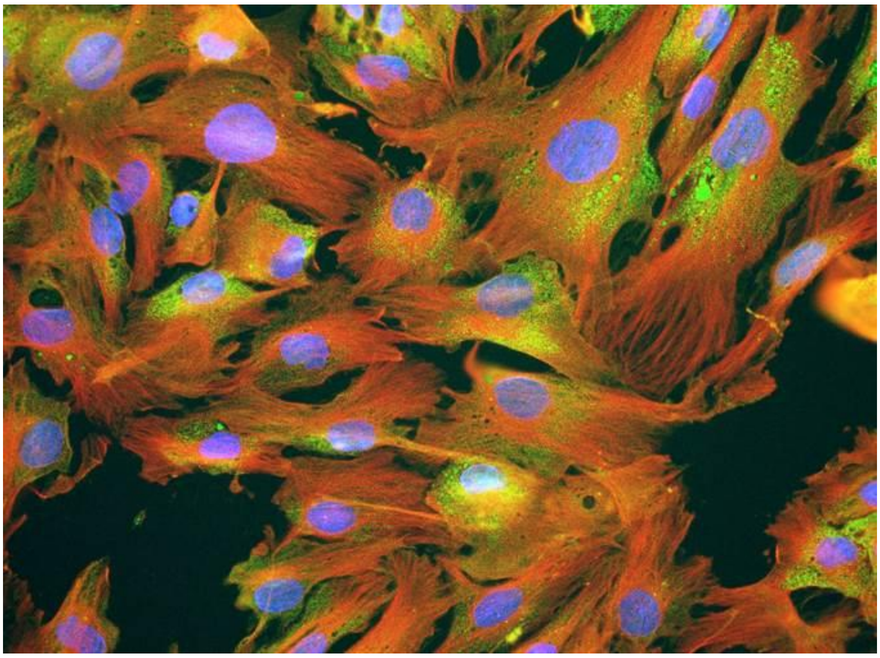

B

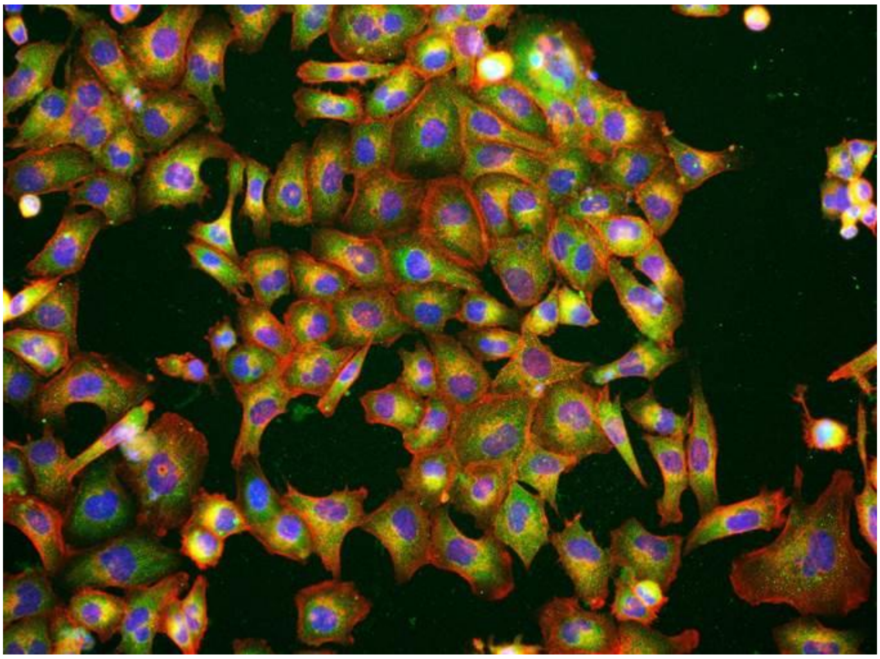

Supplementary Figure 2

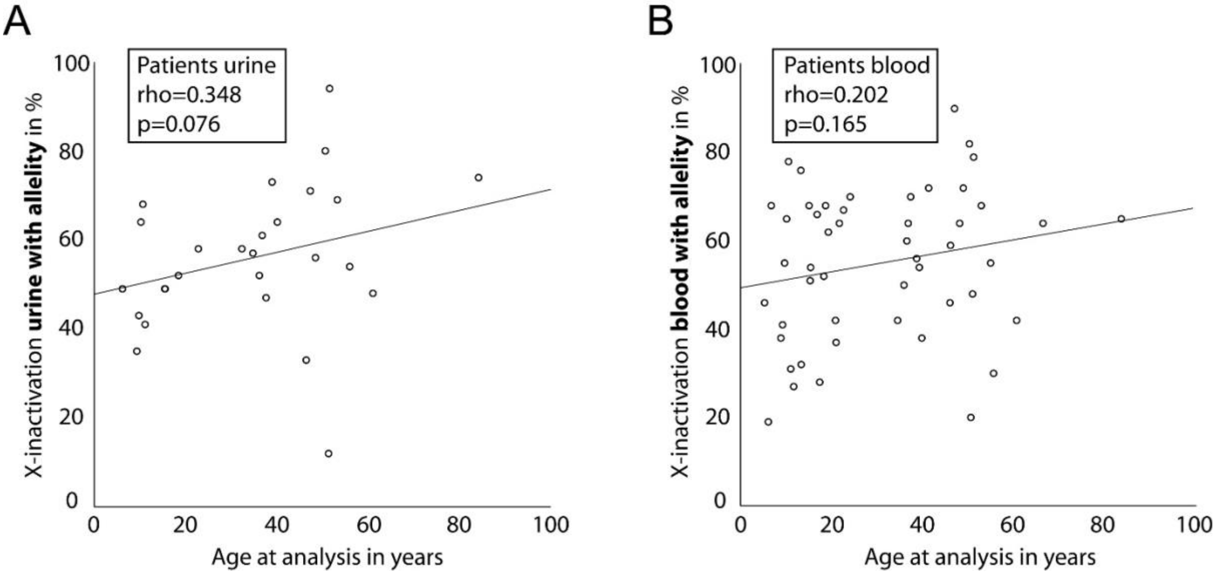

Supplementary Figure 3

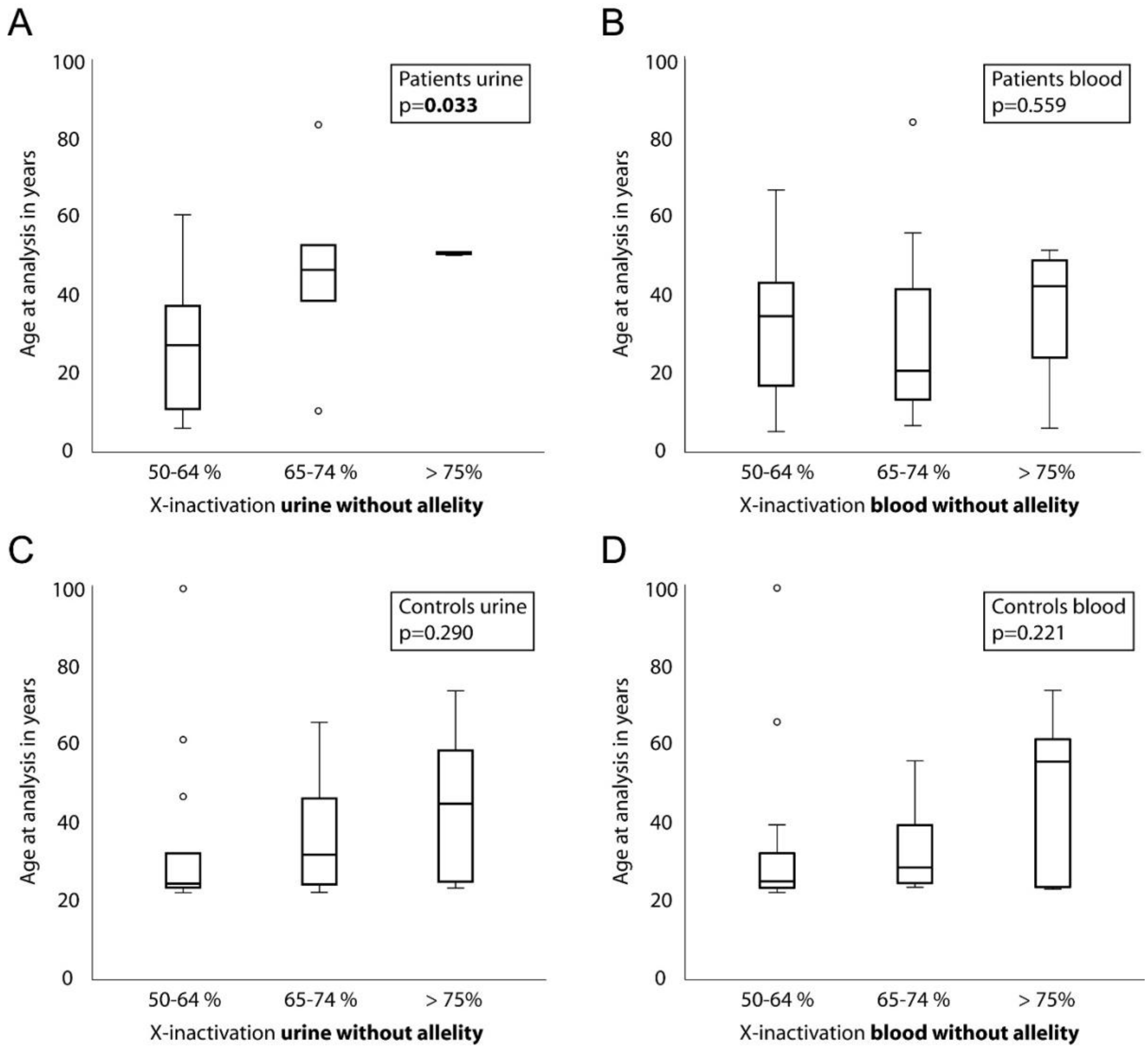

Supplementary Table and Figure Legends

Supplementary Table 1. Overview of identified variants and clinical phenotype of the female individuals with Alport syndrome. ACMG, American College of Medical Genetics; ESKD, end-stage kidney disease; N/A, not applicable.

Supplementary Table 2. Phenotypic characteristics and severity of *COL4A5* variants. §Differing patient numbers: proteinuria (n=53).

Supplementary Table 3. Comparison of X-inactivation in blood and urine with categorical phenotypic markers in individuals with a heterozygous disease-causing variant in *COL4A5*. §Calculation of p-values was not done due to the small number of cases for „yes“ in each category.

Supplementary Figure 1. CD10-staining of podocytes and tubulus cells

Podocytes (A) and tubulus cells (B) were fixed in formalin and stained for CD10 using a species-specific secondary antibody (green), actin filaments showing the cell boundaries (phalloidin, red) and nuclei using diamidinphenylindol (DAPI, blue).

Supplementary Figure 2. Correlation between age of individuals and X-inactivation (with allelity) in blood and urine cells.

Scatter-dot plots of the association of participant age and X-inactivation (with allelity) in blood (A) and urine cells (B) for individuals with a heterozygous disease-causing *COL4A5* variant. Spearman's rho was calculated and is displayed in the boxes. Linear regression lines were plotted for illustration purposes.

Supplementary Figure 3. Comparison of age between groups of X-inactivation skewness

Boxplots for age of individuals compared between different levels of X-inactivation skewness (non-skewed, 50-64%; mildly skewed, 65-74%; skewed, >75%). Data is displayed for individuals with a heterozygous disease-causing *COL4A5* variant (A, B) and healthy controls (C, D).
